# Imaging beta-amyloid (Aβ) burden in the brains of middle-aged individuals with alcohol-use disorders: a [^11^C]PIB PET study

**DOI:** 10.1038/s41398-021-01374-y

**Published:** 2021-05-01

**Authors:** Margaret R. Flanigan, Sarah K. Royse, David P. Cenkner, Katelyn M. Kozinski, Clara J. Stoughton, Michael L. Himes, Davneet S. Minhas, Brian Lopresti, Meryl A. Butters, Rajesh Narendran

**Affiliations:** 1 Department of Radiology, University of Pittsburgh, Pittsburgh, PA USA; 2 Department Psychiatry, University of Pittsburgh, Pittsburgh, PA USA

**Keywords:** Addiction, Hippocampus

## Abstract

No in vivo human studies have examined the prevalence of Alzheimer’s disease (AD) neuropathology in individuals with alcohol-use disorder (AUD), although recent research suggests that a relationship between the two exists. Therefore, this study used Pittsburgh Compound-B ([^11^C]PiB) PET imaging to test the hypothesis that AUD is associated with greater brain amyloid (Aβ) burden in middle-aged adults compared to healthy controls. Twenty healthy participants (14M and 6F) and 19 individuals with AUD (15M and 4F), all aged 40–65 years, underwent clinical assessment, MRI, neurocognitive testing, and positron emission tomography (PET) imaging. Global [^11^C]PiB standard uptake value ratios (SUVRs), cortical thickness, gray matter volumes (GMVs), and neurocognitive function in subjects with AUD were compared to healthy controls. These measures were selected because they are considered markers of risk for future AD and other types of neurocognitive dysfunction. The results of this study showed no significant differences in % global Aβ positivity or subthreshold Aβ loads between AUD and controls. However, relative to controls, we observed a significant 6.1% lower cortical thickness in both AD-signature regions and in regions not typically associated with AD, lower GMV in the hippocampus, and lower performance on tests of attention as well as immediate and delayed memory in individuals with AUD. This suggest that Aβ accumulation is not greater in middle-aged individuals with AUD. However, other markers of neurodegeneration, such as impaired memory, cortical thinning, and reduced hippocampal GMV, are present. Further studies are needed to elucidate the patterns and temporal staging of AUD-related pathophysiology and cognitive impairment. Imaging β-amyloid in middle age alcoholics as a mechanism that increases their risk for Alzheimer’s disease; Registration Number: NCT03746366.

## Introduction

A long-established relationship exists between alcohol use disorder (AUD) and cognitive impairment^1,2^. More recently, evidence supporting an association between AUD and Alzheimer’s disease (AD) risk has begun to accumulate. For example, a recent epidemiological study indicates an approximately twofold elevation in the incidence of AD in individuals with AUD compared to the population at large. This relationship was especially strong when AUD was examined as a risk factor for the onset of dementia in middle-aged adults^3^. In addition, animals fed high ethanol diets demonstrated an upregulation of β-amyloid (Aβ) in the brain parenchyma, one of the pathological hallmarks of AD, as well as its precursor protein (APP) and the secretase enzymes responsible for the cleavage of APP when compared to controls^4,5^.

Although these studies provide compelling evidence for a potential linkage between AUD and AD, other studies challenge the putative relationship between AUD and AD pathology. For instance, a post-mortem study comparing brain tissue from 54 individuals who consumed alcohol heavily and age- and gender-matched controls (age 53 ± 1 years) found no significant elevation in Aβ aggregates, hyperphosphorylated τ, or α-synuclein in the alcohol abusing group^6^. Given the retrospective nature of this study, a number of potential confounding variables may have been present (e.g., survivor bias, psychiatric and medical comorbidities, periods of abstinence from alcohol, and comorbid use of other substances), which complicates the interpretation of these data^7^. Another recent study, in which 414 community members (age 70.9 ± 7.8 years), all without dementia or alcohol-related disorders, underwent carbon-11-labeled Pittsburgh Compound-B ([^11^C]PiB) positron emission tomography (PET) imaging, found no elevation in Aβ accumulation in heavy drinkers (>14 drinks per week) compared to their abstinent counterparts^8^. However, given that the presence of any alcohol-related disorder was an exclusion criterion for this study, it is difficult to extend the interpretation of these findings to individuals with AUD. These studies highlight that, despite considerable interest in characterizing the putative relationship between AUD and AD, the existing data are unable to support a consensus.

Given the animal models and epidemiological data that suggest Aβ deposition in middle-aged adults, and the fact that middle-aged individuals who consume alcohol heavily exhibit neurocognitive impairments without dementia diagnoses^2,9^, we were interested in studying this topic in humans using PET imaging. As the epidemiological data are strongest in middle-aged adults and research demonstrates that some cognitively normal elderly individuals develop Aβ plaques^10,11^, we were interested only in studying middle-aged adults in order to minimize age-related global positivity.

The primary aim of the present study was to use [^11^C]PiB PET imaging^12^ to test the hypothesis that AUD is associated with higher Aβ load in middle-aged AUD subjects compared to healthy controls (HCs). Cortical thickness and hippocampal gray matter volume (GMV), two established magnetic resonance imaging (MRI) outcomes related to AD severity^13^, were also measured in order to evaluate the potential structural abnormalities linking AUD and AD. The secondary aim of this study was to assess whether Aβ load, cortical thickness, or hippocampal GMV were correlated with neurocognitive impairments in AUD.

## Materials and methods

### Study population and clinical assessments

The University of Pittsburgh Human Research Protection Office Institutional Review Board and Radioactive Drug Research Committee approved this study. All subjects provided written informed consent. Twenty healthy participants (14M and 6F) and 19 individuals with AUD (15M and 4F), all aged 40–65 years, completed the study. All subjects were recruited via advertisements in newspaper, bus, and online ads, as well as the University of Pittsburgh research registry (Pitt + Me). HCs were age- and sex-matched to AUD subjects.

The sample size of the study was selected based off of [^11^C]PiB data acquired in *n* = 16 cognitively normal HCs at the University of Pittsburgh, which found 18% (3/16) of individuals to be Aβ+^14^, as well as autopsy studies that suggest Aβ plaques are observed in ~10 to 15% of subjects aged 36–55 years^15^. This, in conjunction with epidemiological data suggesting that within a 5-year period the risk to develop AD is twofold higher in individuals with AUDs compared to controls^3^, suggests that a [^11^C]PiB study of this size should be adequately powered to detect between-group differences in Aβ+.

Inclusion criteria for AUD subjects were as follows: (1) males and females 40–65 years old; (2) fulfill DSM-5 criteria for AUD of at least moderate severity (4+ criteria); (3) no other lifetime Diagnostic and Statistical Manual of Mental Disorders, Fifth Edition (DSM-5) major psychiatric disorders such as schizophrenia, schizoaffective disorder, bipolar disorder, and developmental disorders; (4) no current use (past 4 weeks) of opiates, sedative-hypnotics, cocaine, amphetamines, 3,4-Methylenedioxymethamphetamine (MDMA), and phencyclidine (PCP), as well as moderate to severe cannabis use (i.e., ≥ twice a week); (5) no severe unstable medical or neurological illnesses; (6) no history of cancer within the previous 5 years; (7) not currently pregnant; (8) neither currently employed as a radiation worker nor having participated in radioactive drug research protocols within the previous year such that the total cumulative annual radiation dose would exceed the radiation dose limits specified in the Food and Drug Administration regulations; (9) no metallic objects in the body that are contraindicated for MRI; and (10) no first-degree relative with AD or related dementias. HC subject criteria included the following: (1) males or females between 40 and 65 years old; (2) no present or past history of heavy drinking as defined in Substance Abuse and Mental Health Services Administration criteria (i.e., drinking 5 or more drinks on the same occasion on each of 5 or more days in the past 30 days); (3) criteria 3–10 from the AUD subject group criteria.

Clinical assessments performed included the following: (i) National Institute on Drug Abuse Core: Tier 1 and Tier 2 PhenX Toolkit for collection of individual and family history of substance use and addiction history; (ii) Structured Clinical Interview for DSM-5 for determination of any psychiatric diagnoses; (iii) addiction rating scales for quantification of addiction severity including the Addiction Severity Index^16^, the Substance Use Inventory^17^, the Alcohol Dependence Scale^18^, the Michigan Alcohol Screening Test (MAST)^19^, the Penn Alcohol Craving Scale^20^, and (iv) The Fagerstrom Test for Nicotine Dependence^21^. Participants underwent a physical exam, labs, urine drug screen, and pregnancy test (if female). Subjects’ hair or fingernails and blood were also analyzed for ethyl glucuronide (an alcohol metabolite) and γ-glutamyl transferase (GGT) to supplement self-reported amounts of alcohol consumption^22^. Genotyping for apolipoprotein-E (APOE) was performed on all subjects, as APOE genotype has been shown to influence the age of onset and rate of accumulation of Aβ burden^23,24^, and several [^11^C]PiB PET imaging studies have shown that APOE-ε4 positivity is associated with higher Aβ burden relative to non-carriers^11,25–27^. Genotyping was then considered as a co-variate in data analysis.

### Neurocognitive testing

To assess neurocognitive function, all subjects underwent the Repeatable Battery for the Assessment of Neuropsychological Status (RBANS), selected tasks from the Delis-Kaplan Executive Function System (D-KEFS), the Digit Span and Coding subtests of the Wechsler Adult Intelligence Scale-IV, and the California Verbal Learning Test-II. The RBANS was selected because it assesses several cognitive domains (Immediate and Delayed Memory, Language, Attention, and Visuospatial/constructional ability) and provides a Total Index Score that is a measure of overall neurocognitive function^28^. Included D-KEFS tasks were (1) Color-Word Interference Task, which measures domain ability to inhibit an automatic response; (2) Trail Making Test, which assesses flexibility of thinking; and (3) Verbal Fluency, which measures letter (F, A, S) fluency and semantic (animal) fluency. These D-KEFS tests were then co-normed to compute a mean executive functioning score for each participant.

### Image acquisition

Prior to PET imaging, a magnetization-prepared rapid gradient echo structural MRI scan was obtained using a Siemens 3T Trio scanner for brain region-of-interest (ROI) determination. All AUD subjects were confirmed to have a blood alcohol concentration (BAC) < 0.08 with breathalyzer test prior to MRI. The breathalyzer and the Clinical Institute Withdrawal Assessment for Alcohol–Revised score < 15 were used to confirm that AUD subjects were not intoxicated or in withdrawal prior to PET^29^. The synthesis of [^11^C]PiB was carried out as previously described^30^. PET imaging sessions were conducted using a Siemens ECAT Exact HR + PET scanner (Siemens Healthcare GmbH, Erlangen, Germany) as previously described^31^. [^11^C]PiB was injected as a bolus over 20 s. After a 35 min delay, participants were positioned in the scanner for a 10 min transmission scan. PET emission data were collected over a 20 min period beginning 50 min after injection^32^. Data were constructed using filtered backprojection with Fourier rebinning and standard quantitative data corrections were applied, including those for photon attenuation and scatter, electronics dead time, and radionuclide decay.

### Image analyses

Image analyses were performed by one analyst who was blinded to the group of the participants. MRI and [^11^C]PiB PET data analyses have been described previously^33^. Briefly, MRIs were processed using a FreeSurfer version 5.3^34^ pipeline and atlas. FreeSurfer atlas-derived striatum were substituted for that of the Imperial College London Clinical Imaging Centre Atlas, which demarcates functional subdivisions^35^. [^11^C]PiB PET images were averaged over the 50–70 min post-injection interval, and co-registered and resliced to the space of individual MRIs. Using cerebellar gray matter as a reference region, standard uptake value ratios (SUVR) were determined for nine target ROIs and a global volume-weighted average of Aβ load in these nine regions. Partial volume effects were corrected using the geometric transfer matrix (GTM) method^36^.

Cortical thickness (mm) and GMVs (mm^3^) were derived from FreeSurfer^37^. To create an AD-signature composite ROI, the entorhinal, inferior temporal, middle temporal, and fusiform regions were combined into a surface-area-weighted average^38^. Additional composite surface-weighted ROIs were created for the prefrontal, parietal, and occipital cortices, to provide a regional contrast with the AD-signature composite ROI; these are described further in the Supplement^39^. GMVs were normalized to each subject’s respective FreeSurfer-derived intracranial volume.

### Statistical analysis

Group differences in demographics, alcohol-use characteristics, and laboratory tests were assessed using two-sample *t*-tests, Mann–Whitney *U*-tests, *χ*^2^-tests, and Fisher’s exact tests as appropriate. All tests were two-tailed, with significance thresholds at *p* < 0.05.

Neurocognitive measures were transformed into *Z*-scores using HC subjects’ distributions and clustered into six cognitive functioning domains as follows: attention, immediate memory, delayed memory, visuospatial, language, and executive functioning. Internal consistency of each domain was measured using Cronbach’s *α*. Two-tailed one-sample *t*-tests assessed whether mean domain scores in the AUD group were statistically significant from zero. Group differences in raw scores of individual tests were analyzed as appropriate through the use of two-tailed independent *t*-tests, Mann–Whitney *U*-tests, and analyses of covariance adjusted for age and education. All tests were significant at *p* < 0.05.

As appropriate, two-tailed, two-sample *t*-tests and Mann–Whitney *U*-tests assessed group differences in [^11^C]PiB SUVRs, cortical thickness, and GMV. Significance was set at *p* < 0.006 (*p* = 0.05/9) for regional [^11^C]PiB SUVRs, *p* < 0.013 (*p* = 0.05/4) for subcomponents of the AD-signature cortical thickness ROI, and *p* < 0.007 (*p* = 0.05/7) for GMV per Bonferroni correction. Group differences in regional [^11^C]PiB SUVRs, subcomponents of the AD-signature cortical thickness ROI, and regional GMV were analyzed using linear mixed models (LMMs) with ROI as a repeated measure and diagnostic group (AUD or HC) as a fixed factor; ROI-by-diagnostic group interactions were included as explanatory variables. LMMs were repeated twice: first, with APOE-ɛ4 allele status as an additional fixed factor and, second, with both tobacco use (smoker vs. non-smoker) and tobacco use-by-diagnostic group interaction in the model. Significance for all LMMs was set at *p* < 0.05.

Correlation analyses explored associations of global [^11^C]PiB SUVR, AD-signature composite cortical thickness, and hippocampus GMV with alcohol use, laboratory characteristics, and neurocognitive measures in the AUD group. Spearman’s rank-order correlations were employed due to non-normality of sample distributions. We restricted analyses to these three imaging outcomes because of their relationship to AD severity; significant relationships with other outcomes are described in the Supplement. Correlations were repeated partialing out age. No corrections for multiple comparisons were made for the clinical correlations because they were exploratory. Thus, correlations were considered significant at *p* < 0.05.

Statistical analyses were performed using SAS Software version 9.4 (SAS Institute, Cary, NC) and SPSS version 26 (IBM Corp., Armonk, NY). All SAS or SPSS code is available upon request to the authors.

## Results

### Demographics and clinical characteristics

AUD and control groups were similar with respect to all demographic factors (Table 1). There were no differences in medications or medical comorbidities between groups (complete data in Supplemental Tables 1 and 2). No participants had any current comorbid psychiatric or substance-use disorders. Relative to controls, those with AUD drank more alcohol per use, spent more days drinking alcohol per week, and drank more alcohol per week (*p* < 0.0001 for all three measurements). The AUD group displayed greater concentrations of GGT (*p* = 0.001) and aspartate aminotransferase compared to controls (*p* = 0.01). Groups did not differ by the number of years they had been using alcohol (*p* = 0.30) (Table 1). Vitamins B1, B12, and folic acid levels were statistically similar between groups (Table 1). All AUD subjects had a BAC of 0.00 on PET scan day and no subjects demonstrated clinically significant signs of withdrawal (Table 1).

**Table 1:** Demographics.

	Mean (SD) or *N*		
	Subjects with alcohol-use disorder (*n* = 19)	Healthy controls (*n* = 20)	*p*-Value
Demographics			
Age	55.0 (6.2)	55.0 (6.9)	0.98
Female	4.0	6.0	0.72
Caucasian	18.0	17.0	0.61
Education (years)	15.6 (2.5)	16.4 (1.5)	0.23
BMI	28.8 (5.5)	27.9 (4.2)	0.55
APOE-ɛ4	5.0	3.0	0.38
Tobacco use (Fagerström test for nicotine dependence)	7.0	2.0	0.06
Minimal tobacco use	2.0	2.0	1.0
Moderate tobacco use	3.0	0.0	0.11
High tobacco use	2.0	0.0	0.23
Positive cannabis screening	1.0	0.0	0.49
Comorbid disorders			
Depressive disorders including alcohol-induced (past 12 months)	0.0	0.0	1.0
Depressive disorders including alcohol-induced (prior past 12 months)	3.0	0.0	0.11
Anxiety disorders including alcohol-induced (past 12 months)	0.0	0.0	1.0
Anxiety disorders including alcohol-induced (prior past 12 months)	0.0	0.0	1.0
Cardiovascular disease	2.0	0.0	0.23
Diabetes mellitus	1.0	0.0	0.49
Hypertension	4.0	2.0	0.41
Hypothyroidism	2.0	2.0	1.0
Alcohol-use characteristics			
Michigan Alcohol Screening Test (MAST)	12.5 (4.0)	–	–
Alcohol Dependence Scale (ADS)	18.6 (6.4)	–	–
Penn Alcohol Craving Scale (PACS)	18.2 (5.8)	–	–
Number of DSM-5 AUD Criteria Symptoms	7.8 (1.5)	–	–
Hair ethyl glucuronide >8 pg/mg^a^	11	0	<0.0001
Years of alcohol use	27.4 (13.8)	21.4 (16.1)	0.30
Number of standard drinks per use	11.8 (6.8)	1.3 (1.1)	<0.0001
Number of days drinking per week	5.0 (1.9)	0.9 (0.9)	<0.0001
Number of standard drinks per week	61.8 (52.6)	1.3 (1.7)	<0.0001
BAC (day of PET scan)	0.00 (0.00)	–	–
Clinical Institute Withdrawal Assessment for Alcohol–Revised (CIWA-Ar) (day of PET scan)	0.37 (0.83)	–	–
Laboratory characteristics			
Total bilirubin (mg/dL)	0.6 (0.3)	0.6 (0.3)	0.83
Direct bilirubin (mg/dL)	0.1 (0.1)	0.1 (0.04)	0.35
Alanine aminotransferase (IU/L)	37.8 (35.4)	23.2 (13.1)	0.11
Aspartate aminotransferase (IU/L)	42.2 (31.7)	22.6 (5.0)	0.01
Alkaline phosphate (IU/L)	65.5 (21.4)	63.7 (18.0)	0.78
γ-Glutamyl transferase (IU/L)	108.9 (175.1)	19.2 (11.6)	0.001
Total protein (g/dL)	7.2 (0.4)	7.3 (0.5)	0.53
Albumin (g/dL)	6.5 (9.1)	4.5 (0.31)	0.50
Thyroid stimulating hormone (uIU/mL)	1.9 (0.99)	1.5 (0.6)	0.13
Vitamin B12 (pg/mL)	402.9 (180.8)	439.7 (183.7)	0.77
Folic Acid (ng/mL)	21.8 (27.2)	17.8 (4.9)	0.51
Vitamin B1 whole blood (nmol/L)	145.8 (32.0)	133.9 (27.9)	0.22

^a^Samples not available for four AUD subjects and three HC subjects.

### Neuropsychological performance

Cronbach’s *α* exceeded 0.55 for all domains, except executive (*α* = 0.32) and visuospatial (*α* = 0.31). As a result, three tests from the executive domain (Color-Word Inference: Condition 3, Color-Word Interference: Condition 4, and Trail Making: Condition 4) and one test from the visuospatial domain (RBANS Line Orientation) were chosen for further analyses.

One-sample *t*-tests revealed that, in the AUD group, mean scores in attention (*p* = 0.01), immediate memory (*p* = 0.001), and delayed memory (*p* = 0.001) domains differed from 0, indicating worse performance in all three domains relative to HC. Two-sample tests detected that, within the attention domain, those with AUD performed worse on Trail Making Test Time (*p* = 0.01) and RBANS Coding (*p* = 0.02). Within immediate memory, the AUD group scored worse on CVLT List A Trials 1–5 (*p* = 0.02), CVLT Short Delay Recall (*p* = 0.01), and RBANS List Learning (*p* = 0.01). In the delayed memory domain, the AUD group performed worse on RBANS Story Recall (*p* = 0.003). The AUD group additionally scored lower in Every Day Cognition (*p* = 0.03). These findings persisted after adjusting for age and education (Table 2).

**Table 2:** Neurocognitive raw and domain scores.

	Subjects with alcohol-use disorder (*n* = 19)		Healthy controls (*n* = 20)	
	Raw scores	*Z*-scores	Raw scores	*Z*-scores
	Mean (SD)	Mean (SD)	Mean (SD)	Mean (SD)
Modified Mini-Mental State	96.6 (1.6)	0.0 (0.5)	96.6 (3.3)	0.0 (1.0)
Wechsler Test of Adult Reading	41.0 (6.1)	−0.4 (1.3)	42.8 (4.8)	0.0 (1.0)
Every day cognition	19.5 (6.3)	−1.1 (1.9)	15.6 (3.4)^1^	0.0 (1.0)
Attention domain		−0.5 (0.6)		0.0 (0.4)*
WAIS-IV Coding	70.2 (9.4)	−0.2 (0.8)	73.1 (13.0)	0.0 (1.0)
WAIS-IV Digit Span: forward	11.3 (2.0)	−0.2 (0.9)	11.7 (1.8)	0.0 (1.0)
WAIS-IV Digit Span: backward	9.4 (2.5)	−0.2 (1.1)	9.8 (2.2)	0.0 (1.0)
Color-Word Interference: naming time	31.7 (5.6)	−0.7 (1.2)	28.5 (4.7)	0.0 (1.0)
Color-Word Interference: reading time	22.7 (4.1)	−0.4 (1.3)	21.4 (3.1)	0.0 (1.0)
Trail Making Condition 5 Time	34.2 (10.1)	−1.0 (1.3)	26.2 (7.9)*	0.0 (1.0)
RBANS Digit Span	12.2 (2.3)	−0.1 (0.9)	12.4 (2.5)	0.0 (1.0)
RBANS Coding	45.9 (8.6)	−0.8 (1.0)	52.8 (8.4)*	0.0 (1.0)
Immediate memory domain		−0.7 (0.8)		0.0 (0.8)*
CVLT-II List A Trials	45.8 (11.2)	−0.7 (0.9)	54.7 (11.9)*	0.0 (1.0)
CVLT-II Short Delay Recall	8.2 (4.1)	−0.9 (1.1)	11.6 (3.7)*	0.0 (1.0)
RBANS List Learning	25.4 (4.3)	−0.7 (0.8)	29.6 (5.7)*	0.0 (1.0)
RBANS Story Recall	18.1 (3.1)	−0.5 (1.0)	19.7 (3.2)	0.0 (1.0)
Delayed memory domain		−0.6 (0.7)		0.0 (0.7)*
CVLT-II Long Delay Recall	8.2 (4.4)	−0.5 (0.9)	10.8 (4.8)	0.0 (1.0)
RBANS List Learning Recall	4.5 (2.8)	−0.6 (1.0)	6.2 (2.8)	0.0 (1.0)
RBANS Story Recall	8.7 (2.3)	−1.1 (1.3)	10.6 (1.7)*	0.0 (1.0)
RBANS Figure Recall	13.4 (3.7)	−0.2 (1.1)	13.9 (3.3)	0.0 (1.0)
Language domain		−0.1 (0.5)		0.0 (0.8)
RBANS Picture Naming	10.0 (0.0)	0.2 (0)	10.0 (0.2)	0.0 (1.0)
RBANS Semantic Fluency	19.5 (5.2)	−0.3 (0.9)	21.5 (5.7)	0.0 (1.0)
FAS total	43.7 (11.5)	0.1 (1.0)	42.7 (11.5)	0.0 (1.0)
Animals	20.5 (4.8)	−0.3 (0.8)	22.1 (5.9)	0.0 (1.0)
Executive functions				
Color-Word Interference: Condition 3	11.4 (2.1)	0.1 (0.8)	11.2 (2.5)	0.0 (1.0)
Color-Word Interference: Condition 4	11.2 (2.7)	−0.2 (0.9)	11.7 (3.1)	0.0 (1.0)
Trail Making Test: Condition Time 4	90.3 (26.5)	−0.2 (0.6)	82.1 (43.6)	0.0 (1.0)
Visuospatial functions				
RBANS Line Orientation	15.1 (5.0)	−1.4 (2.3)	17.2 (2.3)	0.0 (1.0)

*p < 0.05

### [^11^C]PiB standard uptake value ratio

Subjects did not differ by injected dose (AUD = 17.0 ± 1.2 mCi; HC = 17.3 ± 1.9 mCi, *p* = 0.65) or injected mass (AUD = 2.6 ± 0.9 μg; HC = 2.4 ± 1.0 μCi, *p* = 0.50). There were no between-group differences in cerebellar reference region radiotracer retention (*p* = 0.22; see Supplemental Materials). No subject reached the threshold for global [^11^C]PiB SUVR positivity based on thresholds determined for our analysis pipeline (global SUVR > 1.35)^14,33^. After GTM correction, one AUD subject was characterized as globally [^11^C]PiB positive (global GTM-corrected SUVR > 1.73). Two-sample *t*-tests detected no group differences in SUVR for any of the nine target ROIs or the global composite index (Table 3). LMMs found no group differences in either regional uncorrected or GTM-corrected [^11^C]PiB SUVRs regardless of inclusion of APOE e4 allele status (see Supplement). However, in GTM-corrected [^11^C]PiB data, there was a significant main effect for tobacco use in regional differences, but its interaction with diagnosis was not significant (effect of diagnosis: *F*_(1,46)_ = 0.1, *p* = 0.77; effect of region: *F*_(8,69)_ = 69.9, *p* < 0.0001; region-by-diagnosis interaction: *F*_(8,69)_ = 1.1, *p* = 0.38; effect of tobacco use: *F*_(1,50)_ = 16.8, *p* < 0.0001; and tobacco use-by-diagnosis interaction: *F*_(8,50)_ = 0.2, *p* = 0.88). It should be noted that inclusion of tobacco use in the model balanced the data such that group differences in GTM-corrected regional [^11^C]PiB SUVRs were closer to zero (effect of diagnosis: *F*_(1,37)_ = 1.2, *p* = 0.28; effect of region: *F*_(8,70)_ = 70.5, *p* < 0.001; and region-by-diagnosis interaction: *F*_(8,70)_ = 1.1, *p* = 0.39). Results of the LMMs are further described in the Supplement.

**Table 3:** [^11^C]PiB SUVRs.

	Mean (SD)					
	Subjects with alcohol-use disorder (*n* = 19)	Healthy controls (*n* = 20)				
			95% CI	*p*-Value	*d*	Bayes factor (*B*_10_)^a^
SUVR						
Global	1.10 (0.06)	1.10 (0.04)	(−0.02, 0.02)	0.94	0.004	4.3
Anterior cingulate	1.17 (0.10)	1.17 (0.08)	(−0.04, 0.04)	0.72	0.02	4.3
Anterior ventral striatum	1.09 (0.12)	1.11 (0.08)	(−0.05, 0.08)	0.59	0.18	3.7
Superior frontal	1.10 (0.10)	1.09 (0.07)	(−0.03, 0.03)	0.83	0.12	4.0
Orbitofrontal	1.14 (0.08)	1.13 (0.05)	(−0.03, 0.04)	0.99	0.15	3.9
Insula	1.13 (0.06)	1.12 (0.05)	(−0.04, 0.03)	0.70	0.12	4.0
Lateral temporal	1.07 (0.04)	1.07 (0.03)	(−0.02, 0.03)	0.80	0.08	4.1
Parietal	1.07 (0.05)	1.08 (0.04)	(−0.02, 0.04)	0.62	0.16	3.8
Posterior cingulate	1.16 (0.06)	1.19 (0.06)	(−0.01, 0.07)	0.09	0.55	1.2
Precuneus	1.16 (0.07)	1.18 (0.06)	(−0.02, 0.06)	0.42	0.26	3.2
SUVR, GTM-corrected						
Global	1.33 (0.18)	1.26 (0.07)	(−0.02, 0.10)	0.16	0.45	1.8
Anterior cingulate	1.38 (0.22)	1.33 (0.17)	(−0.08, 0.14)	0.77	0.23	3.4
Anterior ventral striatum	0.99 (0.19)	1.02 (0.17)	(−0.08, 0.15)	0.55	0.19	3.6
Superior frontal	1.40 (0.26)	1.31 (0.12)	(−0.03, 0.14)	0.19	0.41	2.1
Orbitofrontal	1.42 (0.18)	1.37 (0.08)	(−0.05, 0.09)	0.69	0.30	2.9
Insula	1.10 (0.12)	1.06 (0.08)	(−0.03, 0.07)	0.51	0.38	2.3
Lateral temporal	1.26 (0.10)	1.22 (0.07)	(−0.09, 0.02)	0.23	0.39	2.2
Parietal	1.33 (0.20)	1.26 (0.09)	(−0.18, 0.02)	0.13	0.51	1.4
Posterior cingulate	1.23 (0.15)	1.21 (0.09)	(−0.06, 0.07)	0.81	0.12	4.0
Precuneus	1.30 (0.21)	1.22 (0.08)	(−0.04, 0.13)	0.27	0.49	1.6

^a^The likelihood ratio of observing this data under the assumption that AUD does not influence amyloid-β vs. the assumption that AUD influences amyloid-β, where *B*_10_ = 1 indicates no evidence in favor of either the null or alternative hypotheses (*B*_10_ = 3–10: moderate evidence that AUD does not influence amyloid-β production). For more information, see Lee and Wagenmakers^59^. Calculated using “Bayesian Statistics” feature in SPSS.

### Cortical thickness

Compared to HC subjects, AUD subjects displayed a 6.1% reduction in cortical thickness in the AD-signature composite ROI. Of the regions comprising the composite ROI, AUD subjects displayed a 5.4% reduction in the inferior temporal gyrus, a 7.1% reduction in the middle temporal gyrus, and a 5.8% reduction in the fusiform gyrus, all of which were significant after Bonferonni correction (Table 4). LMMs similarly detected cortical thickness differences between AUD subjects and controls in the individual regions of the AD-signature ROI (effect of diagnosis: *F*_(1,36)_ = 9.6, *p* = 0.004; effect of region: *F*_(3,58)_ = 101.2, *p* < 0.001; and region-by-diagnosis interaction: *F*_(3,58)_ = 0.6, *p* = 0.62). Inclusion of APOE e4 allele status did not change the results (see Supplemental Materials). Composite ROIs of the prefrontal, parietal, and occipital cortices were also significantly smaller in AUD subjects compared to controls (Table 4).

**Table 4:** MRI outcomes.

	Mean (SD)		
	Subjects with alcohol-use disorder (*n* = 19)	Healthy controls (*n* = 20)	*p-*Value
Cortical thickness (mm)			
AD-signature composite	2.6 (0.2)	2.8 (0.1)	0.0003
Entorhinal	3.5 (0.4)	3.7 (0.4)	0.12
Inferior temporal	2.6 (0.2)	2.8 (0.1)	0.01
Middle temporal	2.6 (0.2)	2.8 (0.1)	0.0001
Fusiform	2.6 (0.2)	2.7 (0.1)	0.002
Prefrontal	2.3 (0.1)	2.4 (0.1)	0.01
Parietal	2.0 (0.1)	2.2 (0.1)	0.0001
Occipital	1.9 (0.1)	2.0 (0.1)	0.003
GM volumes^a^ (unitless)			
Hippocampus	26.4 (3.1)	29.3 (2.2)	0.002
Amygdala	10.6 (1.3)	11.5 (0.9)	0.02
Thalamus	43.0 (4.5)	46.2 (4.3)	0.03
Caudate	22.3 (2.5)	22.2 (2.2)	0.83
Putamen	33.7 (4.0)	36.1 (3.6)	0.05
Nucleus accumbens	4.0 (0.7)	4.4 (0.5)	0.03
Cerebellar cortex	296.6 (30.3)	321.4 (31.5)	0.02

^a^Normalized to ICV. Raw volumes in Supplemental Table 6.

### Gray matter volumes

Compared to controls, subjects with AUD displayed significantly smaller GMV in the hippocampus. Other regions failed to survive Bonferroni correction (Table 4). Results of LMM additionally revealed significant between-group differences in regional GMV (see Supplemental Materials).

### Relationships between clinical variables and imaging outcome measures in AUD

Correlation analyses revealed a positive association between global [^11^C]PiB SUVR and years of alcohol use (*p* = 0.5, *p* = 0.02) (Supplemental Table 3). However, this association did not survive correction for age (*p* = 0.4, *p* = 0.08) (Supplemental Table 4).

Significant negative correlations were detected between AD-signature composite cortical thickness and MAST (*p* = −0.5, *p* = 0.02) (Supplemental Table 3 and Supplemental Fig. 1), and this relationship remained significant after adjusting for age (*p* = −0.5, *p* = 0.03) (Supplemental Table 4). AD-signature composite cortical thickness was additionally correlated with the Color-Word Interference Condition 4 (*p* = −0.7, *p* = 0.001) (Supplemental Table 3), which also survived age-adjustment (*p* = −0.7, *p* = 0.001) (Supplemental Table 4); this correlation was also present in the four subregions (see Supplemental Results). There were no other significant relationships between any of the neuropsychological outcome variables and the three primary imaging outcome measures (see Supplemental Tables 3 and 4).

Significant negative correlations were also detected between hippocampal GMV and GGT (*p* = −0.6, *p* = 0.01) (Supplemental Table 3 and Supplemental Fig. 2), which were unchanged following adjustment for age (*p* = −0.6, *p* = 0.01) (Supplemental Table 4). Similar relationships were also present in the thalamus and nucleus accumbens (see Supplemental Materials).

Significant age-adjusted correlations of clinical and neuropsychological data with other imaging outcomes are included in the Supplement.

## Discussion

The present study found no Aβ-positive subjects in either group without GTM correction. GTM correction led one out of 20 (5%) subjects in the AUD group (and none in the HC group) to be classified as Aβ positive, which is well within the expected 5–15% range for Aβ positivity in neurocognitively normal middle-aged individuals^11^. In addition, there were no significant differences in [^11^C]PiB SUVR between groups to indicate that subthreshold Aβ burden may be higher in the AUD group compared to the HC group. However, this study did find significant lower cortical thickness in individuals with AUD compared to HC both in AD-signature ROI (entorhinal, inferior temporal, middle temporal, and fusiform regions) and also in ROI not typically associated with AD (prefrontal, parietal, and occipital cortices). In addition, GMV in the hippocampus was significantly lower in the AUD group than in the HC group. Significant deficits in attention, as well as immediate and delayed memory in individuals with AUD were also found, even after controlling for subject age and education. Relationships linking the deficits in immediate memory with cortical thinning in the AD-signature composite region and GMV loss in the hippocampus in the AUD group remained at trend level (see Supplemental Tables 3 and 4). Consistent with the notion that elevated alcohol consumption underlies cortical thinning and GMV loss, we found a negative correlation between MAST scores and composite cortical thickness, and a negative correlation between GGT and hippocampal GMV.

Our failure to observe significant differences in both global and regional [^11^C]PiB SUVR values between AUD and HC subjects is consistent with and extends earlier reports showing no elevation in Aβ accumulation in heavy drinkers compared to their abstinent counterparts^8^, as well as with previous post-mortem data that found no significant elevation in aggregation of Aβ in alcohol abusing individuals compared to HCs^6^. The cortical thinning and loss of hippocampal GMV observed in this study are also consistent with previous research in subjects with AUD^40–44^ and of a magnitude such that they are likely clinically significant. Specifically, individuals with AD have demonstrated cortical thinning of 3.7% to 6.5% compared to HC^45^ and asymptomatic healthy subjects who went on to develop AD have been shown to have a 4.0% lower cortical thickness than their peers who did not develop AD^46^. These findings suggest that cortical thinning of the magnitude we observed in AUD subjects in the present study (6.1%) equals or exceeds the degree of thinning observed in mild cognitive impairment (MCI) and AD, and likely would result in detectible cognitive abnormalities such as those we observed in our AUD cohort. The smaller hippocampus GMV we observed in the AUD group is likely to be clinically significant as well. Previous research found that hippocampal GMV was between 7% and 10% smaller in MCI subjects compared to cognitively normal controls^47,48^, which is nearly identical to the 10% between-group difference observed in this study.

Prior studies of cognition in AUD have demonstrated both memory^49^ and attention deficits^2,50,51^ consistent with our findings. However, previous studies in AUD have shown deficits in other neurocognitive domains—such as language and executive function—which were not observed in our study^2,9^. Therefore, our findings are partially consistent with those that have been previously reported. None of the subjects in this study were deficient in vitamin B1, B12, folate, or thyroid stimulating hormone, indicating that other reversible causes of cognitive dysfunction do not underlie the findings of this study. Given that all AUD subjects had a BAC of 0.00 on PET scan day, the deficits we observed are not due to active intoxication but it is possible that some of them are related to the fact that none of the participants had been abstinent for an extended period of time. Currently, the data on whether or not an improvement in neurocognitive functioning would be expected with prolonged abstinence is equivocal, with some studies showing structural brain normalization with abstinence^52^ and partial resolution of deficits with long (>1 year) periods of abstinence, whereas others show the persistence of deficits with long-term abstinence^2,9^. Future studies should scan abstinent individuals with AUD, so that it could be better determined if these findings persist without alcohol use.

These findings suggest that the epidemiologic association between AUD and AD in middle-aged individuals with AUD compared to HCs is not explained by a direct influence of alcohol intake on amyloidogenic processes in a manner that alters amyloid trajectories, as suggested by preclinical studies^4,5^. However, our study does indicate robust cortical thinning in regions associated with neurodegeneration in AD and their corresponding cognitive functions, as well as thinning more broadly throughout the neocortex.

Studies examining the spatial and temporal relationships between Aβ accumulation and cortical thinning in AD have found that Aβ accumulation precedes significant atrophy in the AD-signature region^45,53,54^. At least one report also suggests that Aβ accumulation likely precedes GMV loss^55^. Therefore, given that no significant Aβ accumulation was observed in the present study, the cortical thinning, lower hippocampal GMV, and neurocognitive dysfunction we observed are likely mediated by neurodegenerative disease processes related to ethanol toxicity that are distinct from AD. Supporting this interpretation, several previous studies have demonstrated lower global cortical thickness in individuals with AUD compared to HC, a pattern of thinning that is distinct from that typically observed in AD^40–43,56,57^. This distinct pattern of global thinning is consistent with our findings of 5–8% thinning in cortical regions that are not typically associated with the AD-signature pattern of thinning (Table 4). Furthermore, the significant negative correlation between executive function (as measured by the Color-Word Interference: Condition 4) and composite cortical thickness (Supplemental Table 3) is a relationship that has previously been shown to be dependent on cortical regions that are not necessarily unique to AD, but involved in normal aging^58^.

If this neurodegeneration, distinct from AD, is true and taken in conjunction with the neurocognitive findings of this study, it seems possible that research that relies on a clinical diagnosis of AD—such as the epidemiological study cited previously^3^—may represent an overdiagnosis of AD compared to the true prevalence in AUD populations, as AUD-related neurocognitive dysfunction may be misdiagnosed as AD. Alternatively, the neurodegeneration associated with AUD could lower brain reserve, therefore resulting in accelerated cognitive decline in those individuals with AUD who are predisposed to develop AD, thus resulting in detection of AD at an earlier age. Further research characterizing Aβ accumulation in AUD subjects over 65 years of age is necessary to clarify these issues. In the meantime, the results of this study underscore the need for clinicians to consider Aβ imaging in patients with AUD prior to diagnosing them with AD in the clinic.

Limitations of this study include the fact that subjects were relatively young to reach the threshold of global PiB positivity, AUD subjects were not required to abstain for an extended period of time prior to imaging, and the inclusion of fewer tobacco users in the control group. Additionally, subjects lacked an arterial input to quantitate [^11^C]PiB binding and exclude the presence of between-group differences in Aβ in the cerebellum, which was the reference region. However, statistical analyses revealed no difference in radiotracer uptake in the cerebellum, thus providing high confidence that amyloid burden did not vary significantly between groups (see Supplemental Materials). It should also be noted that the sample size was fairly small. For this reason, the possibility of a Type II error for [^11^C]PiB SUVR findings cannot be ruled out. To exclude this possibility, i.e., to demonstrate that the relatively modest 5% elevation in Global PiB SUVR in AUD subjects is statistically significant would have required us to scan a minimum of 79 subjects/group (see Table 3 that shows the effect size for GTM-corrected PiB SUVR is 0.45).

However, recruiting such a large number of subjects was beyond the scope of this preliminary study. It is also possible that a larger sample size would produce the same null findings, as the difference we detected in global GTM-corrected [^11^C]PiB SUVR was most likely driven by the one subject who surpassed the threshold for global positivity. The distribution-free effect size (*r*) corroborates this point; when accounting for the non-normality in the distribution of global GTM-corrected [^11^C]PiB SUVR, the effect size is nearly two times smaller than that of Cohen’s d (*r* = 0.23; See Supplemental Table 5 for *r* in other non-normal regions). Despite this, our secondary findings for group differences in cortical thickness and GMV are quite strong, and thus are not limited by the small sample size.

In conclusion, we used [^11^C]PiB and PET imaging to evaluate Aβ accumulation in middle-aged adults and found no significant differences in Aβ load between AUD and HC. However, we did find significant cortical thinning, lower hippocampal GMV, and neurocognitive deficits in the AUD group. This indicates that a neurocognitive process distinct from AD is occurring and may precede or underlie future Aβ accumulation in subjects with AUD. Further research is needed to understand the exact nature of these processes.

## Supplementary information

Supplemental Material
