# Supplementary material for: Imaging beta-amyloid (Aβ) burden in the brains of middle-aged individuals with alcohol-use disorders: a [11C]PIB PET study
Source: Transl Psychiatry. 2021 May 1;11:257. doi: 10.1038/s41398-021-01374-y (PMC8088438; doi:10.1038/s41398-021-01374-y)
Supplement: Supplementary file 1 — Supplemental Material [file 41398_2021_1374_MOESM1_ESM.docx]

*Supplemental Table 1. Current medical Comorbidities in individuals with AUD compared to controls.*

|  |  | | **Mean (SD) or N (%)^1^** | | |
| --- | --- | --- | --- | --- | --- |
|  | | **Subjects with Alcohol Use Disorder** | | **Healthy Controls** | |
|  | | **(n = 19)** | | **(n = 20)** | |
| Allergic Rhinitis | | 1.0 (5.3%) | | 0.0 (0.0%) | |
| Arthritis or Other Musculoskeletal Disorders | | 1.0 (5.3%) | | 1.0 (5.0%) | |
| Cardiovascular Disease | | 2.0 (10.5%) | | 0.0 (0.0%) | |
| Dermatitis or Other Skin Disorders | | 2.0 (10.5%) | | 1.0 (5.0%) | |
| Diabetes Mellitus | | 1.0 (5.3%) | | 0.0 (0.0%) | |
| Gastro-Intestinal Dysfunction | | 0.0 (0.0%) | | 1.0 (5.0%) | |
| GERD | | 1.0 (5.3%) | | 1.0 (5.0%) | |
| Hepatic Dysfunction | | 2.0 (10.5%) | | 1.0 (5.0%) | |
| History of Nephrolithiasis | | 0.0 (0.0%) | | 1.0 (5.0%) | |
| History of Pancreatitis | | 1.0 (5.3%) | | 0.0 (0.0%) | |
| History of Peptic Ulcer Disease | | 1.0 (5.3%) | | 1.0 (5.0%) | |
| Hyperlipidemia | | 1.0 (5.3%) | | 1.0 (5.0%) | |
| Hypertension | | 4.0 (21.1%) | | 2.0 (10.0%) | |
| Hypothyroidism | | 2.0 (10.5%) | | 2.0 (10.0%) | |
| Obstructive Sleep Apnea | | 0.0 (0.0%) | | 1.0 (5.0%) | |
| Ophthalmic Disorders | | 1.0 (5.3%) | | 0.0 (0.0%) | |
| Vitamin or Mineral Deficiency | | 4.0 (21.1%) | | 1.0 (5.0%) | |
| ^1^No significant differences in any category | | | | |  |

*Supplemental Table 2. Current medications in individuals with AUD compared to controls.*

|  |  | | **Mean (SD) or N (%)^1^** | |
| --- | --- | --- | --- | --- |
|  | | **Subjects with Alcohol Use Disorder** | | **Healthy Controls** |
|  | | **(n = 19)** | | **(n = 20)** |
| Alcohol Use Relapse Prevention | | 1.0 (5.3%) | | 0.0 (0.0%) |
| Allergy or Asthma Medication | | 2.0 (10.5%) | | 2.0 (10.0%) |
| Anti-Depressant | | 2.0 (10.5%) | | 0.0 (0.0%) |
| Anti-Diabetes Medication | | 1.0 (5.3%) | | 0.0 (0.0%) |
| Anti-Hypertension Medication | | 3.0 (15.8%) | | 2.0 (10.0%) |
| Aspirin | | 3.0 (15.8%)) | | 1.0 (5.0%) |
| Benzodiazepine | | 1.0 (5.3%) | | 0.0 (0.0%) |
| Bowel Regulation | | 0.0 (0.0%) | | 1.0 (5.0%) |
| Glaucoma Medication | | 2.0 (10.5%) | | 0.0 (0.0%) |
| Immunosuppressant | | 1.0 (5.3%) | | 0.0 (0.0%) |
| Muscle Relaxant | | 0.0 (0.0%) | | 1.0 (5.0%) |
| NSAIDs | | 2.0 (10.5%) | | 1.0 (5.0%) |
| Other Supplement | | 2.0 (10.5%) | | 0.0 (0.0%) |
| Proton Pump Inhibitor | | 0.0 (0.0%) | | 1.0 (5.0%) |
| Statin | | 2.0 (10.5%) | | 1.0 (5.0%) |
| Thyroid Medication | | 2.0 (10.5%) | | 2.0 (10.0%) |
| Vitamin or Mineral Supplement | | 6.0 (31.6%) | | 2.0 (10.0%) |
| ^1^No significant differences in any category | | | | |

*Supplemental Table 3.* Spearman rank-order correlations between select subject characteristics and outcome variables in subjects with alcohol use disorders

|  |  | **Rho (*p-*value)** |  | |
| --- | --- | --- | --- | --- |
|  | **Global PiB** | **Composite**  **Cortical Thickness** | **Hippocampal GMV** | |
|  |  |  |  |  |
| *Alcohol Use & Laboratory Characteristics* |  |  |  | |
| Michigan Alcohol Screening Test (MAST) | -0.2 (0.47) | -0.5 (0.02)^1^ | -0.4 (0.07) | |
| Alcohol Dependence Scale (ADS) | 0.002 (0.99) | -0.2 (0.50) | 0.02 (0.95) | |
| Penn Alcohol Craving Scale (PACS) | -0.4 (0.06) | -0.4 (0.12) | -0.1 (0.71) | |
| Number of Alcohol Severity Symptoms | -0.3 (0.15) | -0.01 (0.98) | 0.04 (0.85) | |
| Years of Alcohol Use | 0.5 (0.02)^1^ | 0.2 (0.45) | -0.3 (0.22) | |
| Number of Drinks per Use | -0.1 (0.65) | -0.1 (0.66) | 0.004 (0.99) | |
| Number of Days Drinking per Week (WF) | -0.2 (0.48) | -0.3 (0.17) | 0.1 (0.55) | |
| Number of Drinks per Week (WU) | -0.2 (0.47) | -0.2 (0.18) | 0.03 (0.88) | |
| Gamma-Glutamyl Transferase (IU/L) | 0.3 (0.27) | -0.2 (0.36) | -0.6 (0.01)^1^ | |
|  |  |  |  | |
| *Neurocognitive Functions* |  |  |  | |
| Modified Mini-Mental State | 0.02 (0.91) | -0.3 (0.20) | 0.2 (0.31) | |
| Wechsler Test of Adult Reading | 0.1 (0.68) | -0.2 (0.53) | -0.03 (0.91) | |
| Color Word Interference: Condition 3 | 0.2 (0.50) | -0.2 (0.33) | -0.5 (0.05) | |
| Color Word Interference: Condition 4 | -0.01 (0.95) | -0.7 (0.001)^1^ | -0.3 (0.25) | |
| Trail Making Test: Condition 4 | 0.2 (0.32) | -0.1 (0.82) | 0.4 (0.11) | |
| Attention Domain | 0.2 (0.46) | 0.1 (0.73) | 0.04 (0.88) | |
| Immediate Memory Domain | 0.2 (0.99) | 0.4 (0.10) | 0.4 (0.05) | |
| Delayed Memory Domain | -0.003 (0.99) | -0.1 (0.57) | 0.3 (0.20) | |
| Language Domain | -0.2 (0.50) | -0.2 (0.47) | -0.2 (0.38) | |
| ^1^p<0.05 |  |  | |  |

*Supplemental Table 4.* Spearman rank-order correlations between select subject characteristics and outcome variables following an adjustment for age in subjects with alcohol use disorder.

|  |  | **Rho (*p-*value)** |  |
| --- | --- | --- | --- |
|  | **Global PiB** | **Composite**  **Cortical Thickness** | **Hippocampal GMV** |
|  |  |  |  |
| *Alcohol Use & Laboratory Characteristics* |  |  |  |
| Michigan Alcohol Screening Test (MAST) | -0.3 (0.22) | -0.5 (0.03)^1^ | -0.4 (0.08) |
| Alcohol Dependence Scale (ADS) | -0.09 (0.72) | -0.1 (0.58) | 0.04 (0.88) |
| Penn Alcohol Craving Scale (PACS) | -0.5 (0.04)^1^ | -0.4 (0.13) | -0.1 (0.73) |
| Number of Alcohol Severity Symptoms | -0.3 (0.22) | -0.03 (0.90) | 0.03 (0.91) |
| Years of Alcohol Use | 0.4 (0.08) | 0.3 (0.21) | -0.3 (0.26) |
| Number of Drinks per Use | -0.3 (0.27) | -0.1 (0.80) | 0.04 (0.87) |
| Number of Days Drinking per Week | -0.3 (0.24) | -0.3 (0.21) | 0.2 (0.48) |
| Number of Drinks per Week | -0.4 (0.14) | -0.3 (0.24) | 0.1 (0.76) |
| Gamma-Glutamyl Transferase (IU/L) | 0.2 (0.39) | -0.2 (0.42) | -0.6 (0.01)^1^ |
|  |  |  |  |
| *Neurocognitive Functions* |  |  |  |
| Modified Mini-Mental State | -0.02 (0.94) | -0.3 (0.24) | 0.3 (0.30) |
| Wechsler Test of Adult Reading | 0.1 (0.73) | -0.1 (0.56) | -0.02 (0.92) |
| Color Word Interference: Condition 3 | 0.002 (0.99) | -0.2 (0.44) | -0.5 (0.06) |
| Color Word Interference: Condition 4 | -0.2 (0.53) | -0.7 (0.001)^1^ | -0.3 (0.29) |
| Trail Making Test: Condition 4 | 0.1 (0.26) | -0.01 (0.98) | 0.4 (0.06) |
| Attention Domain | 0.3 (0.23) | 0.04 (0.85) | 0.01 (0.95) |
| Immediate Memory Domain | 0.3 (0.23) | 0.4 (0.13) | 0.4 (0.07) |
| Delayed Memory Domain | 0.1 (0.66) | -0.2 (0.46) | 0.3 (0.24) |
| Language Domain | -0.1 (0.64) | -0.2 (0.42) | -0.2 (0.36) |

^1^p<0.05

*Supplemental Figure 1.* Correlation between AD-signature composite cortical thickness (x-axis) and Michigan Alcohol Screening Test (MAST) scores in subjects with alcohol use disorder.

*
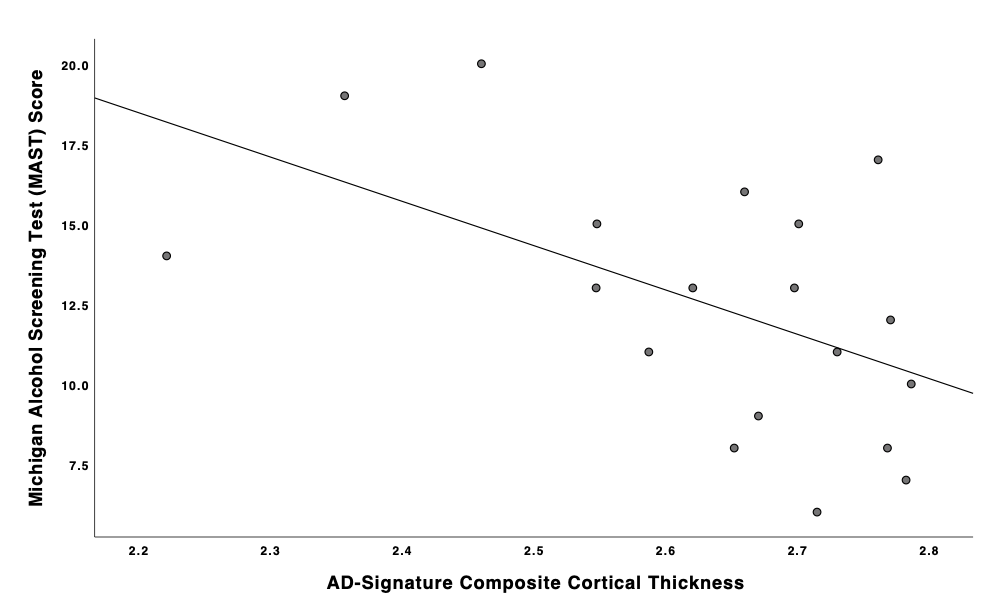
*

*Supplemental Figure 2.* Correlation between hippocampal GMV (x-axis) and gamma-glutamyl transferase (GGT) in subjects with alcohol use disorder. Similar relationships with GGT were observed for thalamus and nucleus accumbens (see supplemental results).

*
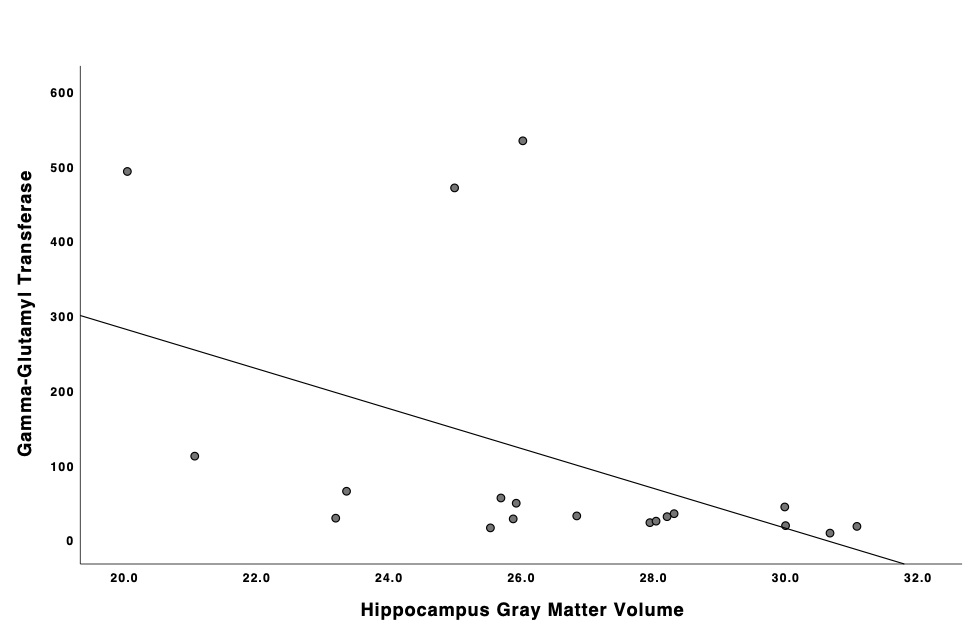
*

*Supplemental Table 5.* Distribution-free effect size (r) of non-normally distributed SUVRs

|  | *r* |
| --- | --- |
| *SUVR* |  |
| Global | 0.01 |
| Anterior cingulate | 0.06 |
| Anterior ventral striatum | -- |
| Superior frontal | 0.03 |
| Orbitofrontal | 0.002 |
| Insula | -- |
| Lateral temporal | -- |
| Parietal | -- |
| Posterior cingulate | -- |
| Precuneus | -- |
|  |  |
| *SUVR, GTM-corrected* |  |
| Global | 0.23 |
| Anterior cingulate | 0.05 |
| Anterior ventral striatum | -- |
| Superior frontal | 0.21 |
| Orbitofrontal | 0.07 |
| Insula | 0.11 |
| Lateral temporal | -- |
| Parietal | -- |
| Posterior cingulate | 0.04 |
| Precuneus | 0.17 |

*Supplemental Table 6.* Raw GM volumes (mm^3^).

|  | **Mean (SD)** | |  | |
| --- | --- | --- | --- | --- |
|  | **Subjects with Alcohol Use Disorder** | **Healthy Controls** | |  |
|  | **(n = 19)** | **(n = 20)** | | ***p-*value** |
| Hippocampus | 4075.5 (477.0) | 4473.7 (437.7) | | 0.01 |
| Amygdala | 1637.2 (206.9) | 1753.7 (182.0) | | 0.07 |
| Thalamus | 6640.4 (877.6) | 7050.0 (782.8) | | 0.13 |
| Caudate | 3463.9 (530.7) | 3387.8 (407.9) | | 0.62 |
| Putamen | 5204.6 (755.1) | 5500.8 (590.8) | | 0.18 |
| Nucleus Accumbens | 615.8 (128.7) | 637.7 (72.3) | | 0.10 |
| Cerebellar Cortex | 45602.4 (3568.0) | 48933.3 (4517.1) | | 0.02 |

**SUPPLEMENTAL ANALYSES AND RESULTS**

**FreeSurfer composite surface-weighted ROIs**

In order to assess cortical thickness in other ROIs associated with cognition, additional composite surface-weighted ROIs were created for the prefrontal cortex (FreeSurfer regions: caudal anterior cingulate, caudal middle frontal, lateral orbitofrontal, medial orbitofrontal, pars opercularis, pars orbitalis, pars triangularis, rostral anterior cingulate, rostral middle frontal, superior frontal, frontal pole) (37), the parietal cortex (FreeSurfer regions: superior parietal, inferior parietal, post central, precuneus, supramarginal), and the occipital cortex (FreeSurfer regions: lateral occipital cortex, cuneus, lingual cortex, pericalcarine).

**Linear Mixed Model Results**

In the LMM that assessed uncorrected regional uncorrected [^11^C]PiB SUVRs, no differences were detected (effect of diagnosis: F_(1,37)_=0.1, p=0.76; effect of region: F_(8,74)_=56.7, p<0.001; region-by-diagnosis interaction: F_(8,74)_=1.6, p=0.14) or regional GTM-corrected [^11^C]PiB SUVRs  (effect of diagnosis: F_(1,37)_=0.1, p=0.76; effect of region: F_(8,74)_=56.7, p<0.001; region-by-diagnosis interaction: F_(8,74)_=1.6, p=0.14). This was also true of the model that assessed regional GTM-corrected [^11^C]PiB SUVRs  (effect of diagnosis: F_(1,37)_=1.2, p=0.28; effect of region: F_(8,70)_=70.5, p<0.001; region-by-diagnosis interaction: F_(8,70)_=1.1, p=0.39)*.*When APOE-ɛ4 allele status was included, no differences were observed by either genotype or uncorrected [^11^C]PiB SUVR (effect of diagnosis: F_(1,31)_=0.03, p=0.86; effect of genotype: F_(1,59)_=2.2, p=0.15; effect of region: F_(8,76)_=56.2, p<0.001; region-by-diagnosis interaction: F_(8,76)_=1.6, p=0.14). This was also true of GTM-corrected [^11^C]PiB SUVR model (effect of diagnosis: F_(1,32)_=1.4, p=0.25; effect of genotype: F_(1,54)_=3.9, p=0.05; effect of region: F_(8,68)_=71.0, p<0.001; region-by-diagnosis interaction: F_(8,68)_=1.1, p=0.37).

The LMM that included APOE-ɛ4 genotype detected cortical thickness differences between AUD subjects and controls in the sub-components of the AD-signature ROI (effect of diagnosis: F_(1,36)_=8.7, p=0.01; effect of genotype: F_(1,65)_=1.5, p=0.22; effect of region: F_(3,58)_=101.3, p<0.001; region-by-diagnosis interaction: F_(3,58)_=0.6, p=0.61).

In terms of regional GMV, LMM detected significant differences between AUD subjects and controls (effect of diagnosis: F_(1,40)_=7.9, p=0.008; effect of region: F_(6,51)_=1300.4, p<0.001; region-by-diagnosis interaction: F_(6,51)_=3.3, p=0.008). Adding APOE-ɛ4 allele status to the model did not attenuate these differences (effect of diagnosis: F_(1,40)_=7.9, p=0.008; effect of genotype: F_(1,55)_=0.6, p=0.45; effect of region: F_(6,51)_=1311.5, p<0.001; region-by-diagnosis interaction: F_(6,51)_=3.3, p=0.008). Further, when tobacco use and its interaction with diagnosis were added to the model, results did not change (effect of diagnosis: F_(1,40)_=7.1, p=0.01; effect of region: F_(6,51)_=1278.1, p<0.0001; region-by-diagnosis interaction: F_(6,51)_=3.3, p=0.008, effect of tobacco use: F_(1,55)_=0.1, p=0.74; tobacco use-by-diagnosis interaction: F_(1,55)_=1.7, p=0.19).

**Age-adjusted correlation analyses of clinical and neuropsychological outcomes with non-GTM-corrected regional [^11^C]PiB SUVRs in subjects with AUD**

The anterior cingulate cortex (rho=-0.5, p=0.03), anterior ventral striatum (rho=-0.5, p=0.04), and the precuneus (rho=-0.6, p=0.02) [^11^C]PiB SUVRs were all negatively correlated with The Penn Alcohol Craving Scale. The orbitofrontal cortex [^11^C]PiB SUVR was negatively correlated with Number of Alcohol Severity Symptoms (rho=-0.5, p=0.03).

Of the neurocognitive scores, the posterior cingulate cortex [^11^C]PiB SUVR was positively correlated with the attention domain (rho=0.6, p=0.01). Additionally, precuneus [^11^C]PiB SUVR was positively correlated with immediate memory (rho=0.5, p=0.04) and delayed memory (rho=0.5, p=0.02).

**Age-adjusted correlation analyses of clinical and neuropsychological outcomes with cortical thickness in the subcomponents of the AD-signature composite ROI and other composite regions in subjects with AUD**

The entorhinal (rho=-0.5, p=0.04) and middle temporal (rho=-0.5, p=0.03) cortices were negatively correlated with MAST.

In terms of the neurocognitive scores, entorhinal cortical thickness was positively correlated with the immediate memory domain (rho=0.6, p=0.01) and the composite parietal ROI was negatively correlated with immediate memory (rho=-0.5, p=0.04). Additionally, entorhinal (rho=-0.5, p=0.02), inferior temporal (rho=-0.5, p=0.02), middle temporal (rho=-0.8, p=0.0002), and fusiform (rho=-0.6, p=0.02) cortical thicknesses were negatively correlated with Color-Word Interference: Condition 4.

**Age-adjusted correlation analyses of clinical and neuropsychological outcomes with subcortical and cerebellar GMV in subjects with AUD**

The thalamus (rho=-0.7, p=0.002) and nucleus accumbens GMV (rho=-0.6, p=0.01) were negatively correlated with Gamma Glutamyl Transferase.

In the neurocognitive scores, GMV of the caudate was positively correlated with the attention domain (rho=0.5, p=0.04). Amygdala (rho=0.5, p=0.02) and thalamus (rho=0.5, p=0.04) GMV were positively correlated with the immediate memory domain. GMV of the nucleus accumbens was negatively correlated with Color-Word Interference: Condition 3 (rho=-0.7, p=0.001) and Color-Word Interference: Condition 4 (rho=-0.6, p=0.01). GMV of the caudate (rho=0.5, p=0.02) and the putamen (rho=0.5, p=0.03) were positively correlated with Trail Making Test: Condition 4.
